# Myocardial fibrosis reversion via rhACE2-electrospun fibrous patch for ventricular remodeling prevention

**DOI:** 10.1038/s41536-021-00154-y

**Published:** 2021-08-10

**Authors:** Zeping Qiu, Jingwen Zhao, Fanyi Huang, Luhan Bao, Yanjia Chen, Ke Yang, Wenguo Cui, Wei Jin

**Affiliations:** 1 Department of Vascular & Cardiology, Ruijin Hospital, Shanghai Jiao Tong University School of Medicine, Shanghai, PR China; 2 Institute of Cardiovascular Diseases, Shanghai Jiao Tong University School of Medicine, Shanghai, PR China; 3 Department of Orthopaedics, Shanghai Key Laboratory for Prevention and Treatment of Bone and Joint Diseases, Shanghai Institute of Traumatology and Orthopaedics, Ruijin Hospital, Shanghai Jiao Tong University School of Medicine, Shanghai, PR China

**Keywords:** Heart failure, Translational research, Biomedical materials, Implants, Drug delivery

## Abstract

Myocardial fibrosis and ventricular remodeling were the key pathology factors causing undesirable consequence after myocardial infarction. However, an efficient therapeutic method remains unclear, partly due to difficulty in continuously preventing neurohormonal overactivation and potential disadvantages of cell therapy for clinical practice. In this study, a rhACE2-electrospun fibrous patch with sustained releasing of rhACE2 to shape an induction transformation niche in situ was introduced, through micro-sol electrospinning technologies. A durable releasing pattern of rhACE2 encapsulated in hyaluronic acid (HA)—poly(L-lactic acid) (PLLA) core-shell structure was observed. By multiple in vitro studies, the rhACE2 patch demonstrated effectiveness in reducing cardiomyocytes apoptosis under hypoxia stress and inhibiting cardiac fibroblasts proliferation, which gave evidence for its in vivo efficacy. For striking mice myocardial infarction experiments, a successful prevention of adverse ventricular remodeling has been demonstrated, reflecting by improved ejection fraction, normal ventricle structure and less fibrosis. The rhACE2 patch niche showed clear superiority in long term function and structure preservation after ischemia compared with intramyocardial injection. Thus, the micro-sol electrospun rhACE2 fibrous patch niche was proved to be efficient, cost-effective and easy-to-use in preventing ventricular adverse remodeling.

## Introduction

Ischemic heart disease is the first cause of death around the world, attributed to more than 9 million deaths per year^1^. Due to the advancement of intervention therapy for myocardial infarction (MI), now the irreversible initial injury can be mitigated by emergency revascularization. Nonetheless, acute MI can evoke structure, electrophysiology and metabolic changes within ventricle. Changes were defined as ventricular remodeling afterwards, which often lead to chronic heart failure and finally cardiovascular death^2^. So now the clinical challenge after MI becomes restricting remodeling, conserving cardiac function and preventing heart failure. Neurohormonal blockade is the foundational therapeutic approach to prevent post-MI remodeling. Regardless of the optimal guideline-directed medical therapy, angiotensin (Ang) II formation cannot be entirely prevented in patients, presumably through angiotensin-converting enzyme independent pathway^3^. For countering overactivated renin–angiotensin–aldosterone axis, angiotensin-converting enzyme 2 (ACE2) was discovered as a novel negative regulator. ACE2 can cleave AngII and generate the cardiovascular protective heptapeptide Ang1–7^4,5^. Furthermore, our previous study had revealed ACE2 is a cardinal downstream molecule of catestatin for its pleiotropic cardiovascular protective effects^6^. In preclinical model, it has already been proven that overexpression of ACE2 at the heart can revert AngII-induced myocardial fibrosis, oxidant injury and cardiac dysfunction^7,8^. In preliminary clinical trial, recombinant human ACE2 (rhACE2) was well tolerated by healthy human participants^9^. And a promising result in conversing AngII to Ang1–7 and normalizing elevated plasma AngII level in heart failure patients have been observed^10^.

Despite the effectiveness and safety of rhACE2 for adverse myocardial remodeling have been confirmed, its clinical application is largely restricted by several limitations. To date, the administration route of rhACE2 is mostly intravenous, by which the retention rate at myocardium is unsatisfied. In physiologic condition, ACE2, as a type-1 transmembrane enzyme, exert its enzymatic activity at the heart by tethering ACE2/Ang1–7/MasR locally in a paracrine signaling way, rather than systemic Ang1–7 production^11^. So it is particularly important to ensure sustained rhACE2 in situ activity by a proper dosing method, protecting it from cleavage^12,13^. On the other hand, repetitive systemic rhACE2 administration exposed whole body vasculature to the injected agent, increasing the risk of adverse events like hypersensitivity or hypotension, while only providing limited improvements to local retention^14^. Another major setback is the short dose-independent terminal half-life of rhACE2, which is only about 10 h in vivo. Previous reported in-man pharmacodynamics study showed at least a daily intravenous rhACE2 dose is required to maintain low plasma AngII levels^9^. Due to the paradox of continuous regional existence requirement and rapid degradation, a single intramyocardial injection of rhACE2 can’t resolve the dilemma in translational medicine. Therefore, we proposed these current issues may be mitigated by developing a fibrous patch, which can provide a long-lasting counter AngII niche at the local infarcted region, reversing fibrosis, limiting apoptosis and preventing remodeling.

Plenty different preparations of materials applied epicardial have been tested for reversing cardiac fibrosis after MI, including gene delivery microneedles^15^, 3D printed human-induced pluripotent stem cell-derived patch^16^, cardiac extracellular matrix loaded hydrogel^17^, extracellular matrix encapsulating secreted factors from cardiac stromal cells^18^, and so on. These materials have proven their effectiveness in preserving heart function after ischemic injury. However, all mentioned materials based their regional therapeutic effects on cell or gene therapies, which naturally came with some limitations. First, materials containing live cells have high demands for storage conditions and limit preservation time in order to retain viability and functionality before application^19^. Also, a carrier derived from animal extracellular matrix is extremely expensive due to complex process, low yield, and poor accessibility^20^. Therefore, it will be hard to become a widely adapted, standardize clinical product. More importantly, we must take the precaution of immune rejection reaction and consequence of uncontrollable proliferation when allogeneic stem cells or gene transcription are involved^21,22^, since all these adverse effects can be lethal. Consequently, these unpredictable risks hampered the translational prospects. Thus, for a more practical choice, we conceived a novel synthesized biomaterial based on mature techniques, like an electrospun fibrous patch, loading a bioactive peptide which targets a key pathophysiologic pathway. By carrying rhACE2 inside a fibrous core, it also solves the dosing challenge of providing continuous in situ rhACE2 activity.

**Fig. 1:**
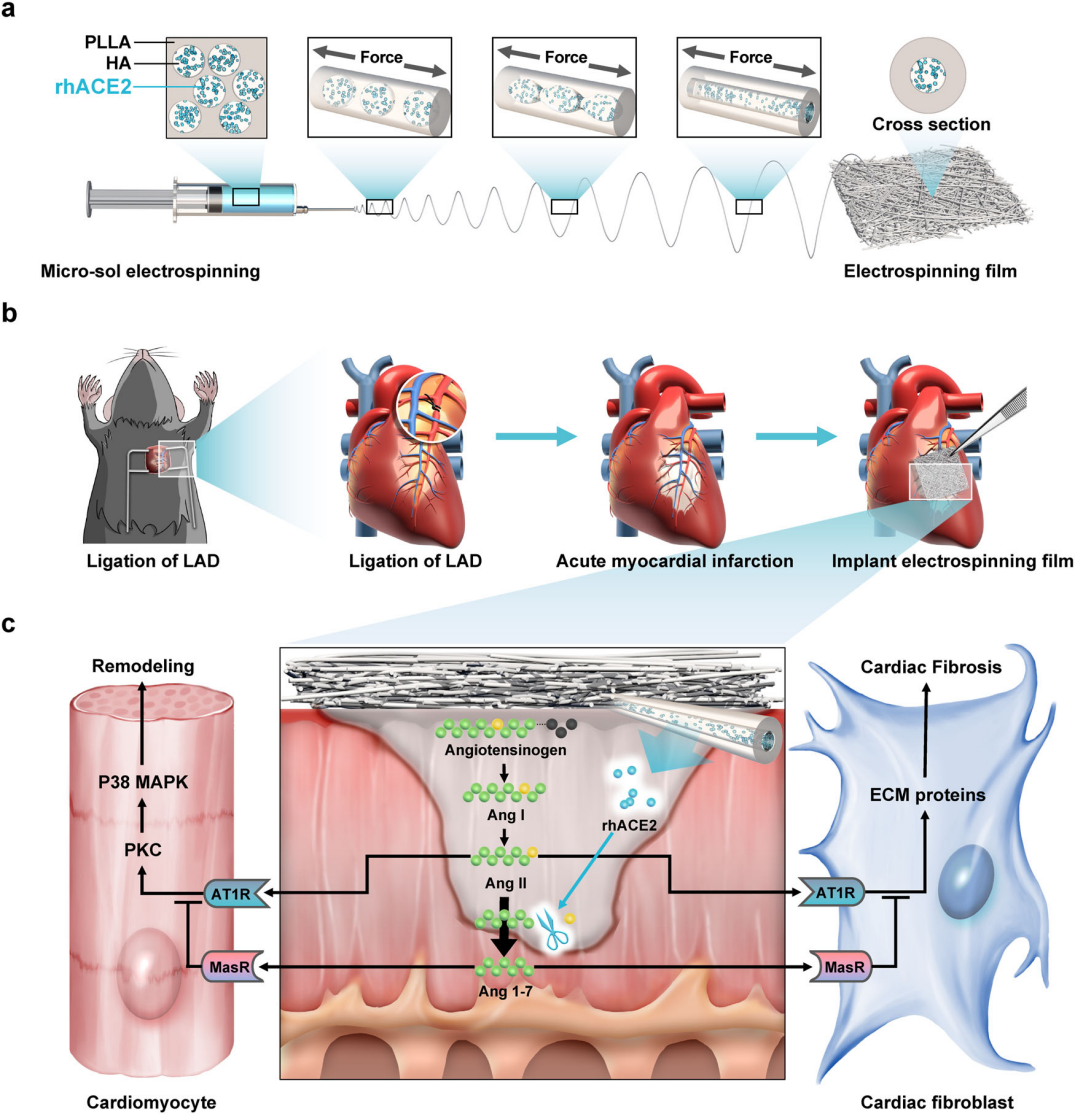
Schematic Illustration. **a** Preparation of rhACE2-loaded electrospun nanofiber patch by hyaluronan (HA) micro-sol electrospun and the mechanism of formation of core-shell structure. **b** Myocardial infarction mice model established by precise ligation of the left anterior descending (LAD) coronary artery along with the illustration of rhACE2 patch implantation. **c** In situ rhACE2 patch niche degrading angiotensin II (AngII) into a cardioprotective heptapeptide, Ang1–7, which counter the AngII/AT1R mediated effects by inhibiting cardiac fibrosis and cardiomyocyte apoptosis. PLLA poly(L-lactic acid), rhACE2 recombinant human angiotensin-converting enzyme 2, AT1R angiotensin II receptor type 1, PKC protein kinase C, MAPK mitogen-activated protein kinase, ECM extracellular matrix.

In this study, determined to overcome these forementioned disadvantages, the micro-sol electrospinning technology was introduced to construct a cost-efficient, easily available electrospun fibrous patch, which can continuously release active rhACE2 precisely into area at risk to reverse post-MI fibrosis and prevent ischemic heart disease. The rhACE2-electrospun fibrous patch introduced here, can induce the AngII dominated pro-fibrosis post-MI niche into an Ang1–7 dominated anti-fibrosis niche after implantation. Since fibrous patch are constructed under an electrostatic field in a highly irregular polymer chain network manner, a storage of electrostatic energy always come along. During the implantation procedure, the stored electric energy can make the patch easily attached to the epicardium without further fixation. First, hyaluronic acid (HA) hydrosol was chosen to encapsulate rhACE2, in a colloid in liquid form^23^. Later, an emulsion was formed with the HA hydrosol dispersed in the spinning solution, and these micro-droplets in emulsion were described as micro-sol particles. This system was previously described by us as micro-sol electrospinning method. A core-sheath structure membrane was further fabricated by encapsulating these HA/rhACE2 micro-sol particles into poly(L-lactic acid) (PLLA) fibers during the electrospinning procedure, which we named as PLLA-HA/rhACE2 (Fig. 1a). Then its physical properties were studied before further investigation. Second, the effects of the PLLA-HA/rhACE2 patch on the survival of neonatal rat cardiomyocytes (NRCMs) under hypoxia condition and the sustained-release effect of biologically active rhACE2 by the core-sheath structure were verified in vitro. Finally, the rhACE2 fibrous patch was implanted on a mice permanent left anterior descending (LAD) coronary artery ligation acute MI model to investigate its effectiveness on preserving cardiac function by serial echocardiography in several time points (Fig. 1b). And its ability to reverse cardiac fibrosis was evaluated by histological staining and analyzed of morphometric parameters (Fig. 1c). Altogether, a rhACE2-electrospun fiber patch niche was developed to reverse cardiac fibrosis, counter adverse remodeling and preserve ventricle function after MI.

## Results

### Characteristics of the fibrous patch

**Fig. 2:**
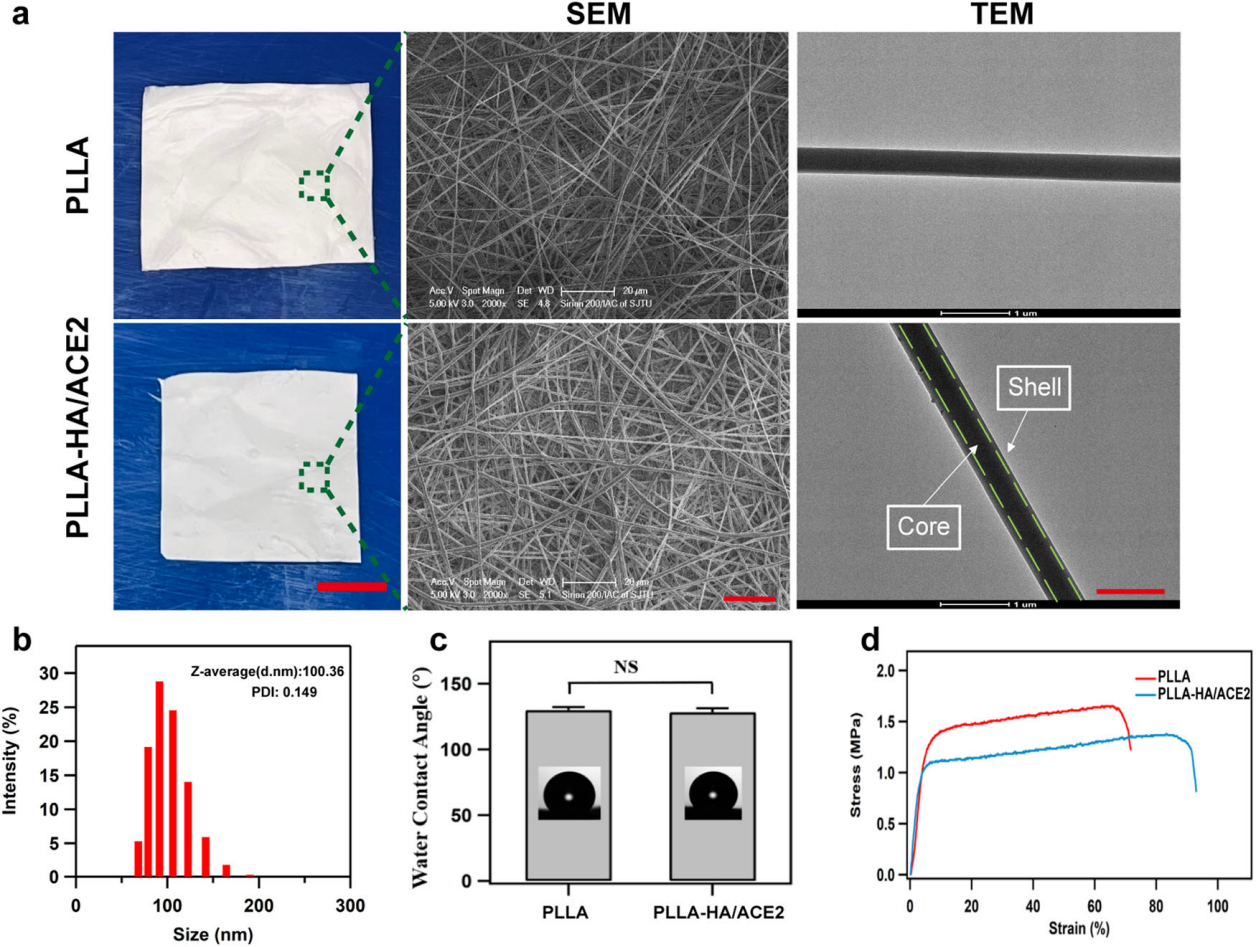
Characterization of the rhACE2 patch. The general view, SEM and TEM micrographs demonstrating the core-shell structure (**a**), dynamic light scattering (**b**), water contact angle (**c**) and stress-strain curve (**d**) from PLLA-HA/ACE2 electrospun nanofibers. Scale bars represent 1 cm in general view, 20 μm in SEM and 1 μm in TEM.

The rhACE2-electrospun fibrous patch was synthesized step by step as we illustrated in Fig. 1a. The morphology features, composition, water contact angle and mechanical properties were characterized separately. Pure PLLA membrane and rhACE2-loaded fibrous patch were successfully fabricated via electrospinning method. For the aspect of surface morphology, a smooth, uniform and randomly oriented appearance could be observed on these electrospun fibers (Fig. 2a). As the TEM image demonstrated, HA/rhACE2 micro-sol particles core was centered in the PLLA-HA/rhACE2 fibers, which was obviously distinct from bare PLLA fibers. The inner diameter of HA/rhACE2 core was measured to be 0.13 ± 0.03 μm. And the average diameters of PLLA and PLLA-HA/rhACE2 fibers were 0.58 ± 0.11 μm and 0.62 ± 0.14 μm. No significant change was observed in diameter between these two fibers.

To identify the size distribution of HA/rhACE2 micro-sol particles dispersion status, DLS was conducted (Fig. 2b). And the results implicated that the mean diameter of micro-sol particles was 100.36 nm, with a poly-dispersity index of 0.149, which demonstrated the consistency of the particles. A water contact angle of 129.98 ± 2.15° and 128.32 ± 2.87° were found for electrospun PLLA and PLLA-HA/rhACE2 patch respectively, showing both groups possess a mild lipophilic trait (Fig. 2c). And no significant difference existed between each other. Stress-strain measurement was tested to explore the mechanical property. The tensile strength of PLLA and PLLA-HA/rhACE2 membrane was 1.65 ± 0.17 and 1.38 ± 0.11 MPa, respectively (Fig. 2d). Compared with pure PLLA membrane, the maximum tensile strength of the PLLA-HA/rhACE2 patch was slightly lower, which could be caused by the addition of a HA core^24^.

### Biocompatibility test

**Fig. 3:**
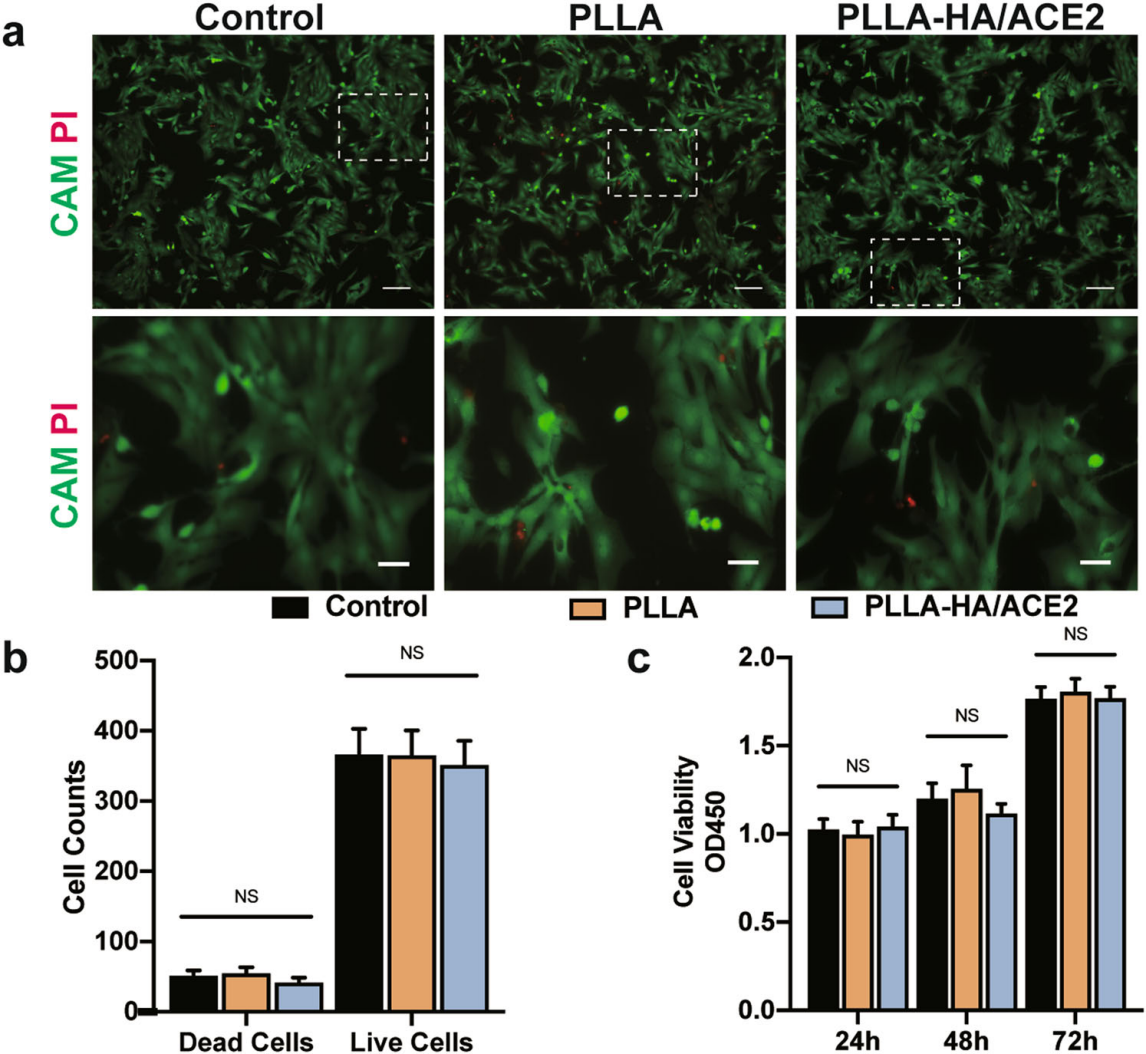
Biocompatibility tests in vitro. **a** Representative live/dead fluorescence stained by Calcein AM (green) and Propidium Iodide (red) at day 3. Scale bars represented 50 and 20 μm for zoomed pictures. **b** Live and dead cell count per HPF of control, PLLA, PLLA-HA/ACE2 groups. *n* = 4/group. **c** CCK-8 cell viability quantification results in different groups after 1, 2, or 3 days. *n* = 4/group. Data were represented as the mean ± SEM and analyzed for statistical significance using One-way ANOVA followed by Tukey’s multiple comparison test; NS no significant difference.

Since PLLA polymers as a biodegradable, biocompatible material has been approved by Food Drug Administration for absorbable biomedical engineering since 2009, safety should be warranted for cell co-culture and in-vivo implantation. In terms of the live/dead cell counts, no significant differences between regular cultured CMs and CMs cultured with PLLA or PLLA-HA/ACE2 group was observed after 72 h (Fig. 3a, b), demonstrating that PLLA nanofiber with or without the internal HA core did not present a cytotoxicity effect. To further illustrate whether PLLA influenced CMs cell viability, a CCK-8 assay was performed after the live/dead studies. The OD_450_ value showed no statistic difference among all three groups after 24, 48 or 72 h co-culture and the growth trend for 3 days preserved in patch co-cultured groups compared with control group (Fig. 3c), indicating the nanofibrous patch didn’t affect in vitro CMs survival. All data were consistent with expectation, demonstrating the nanofibrous patch presented here was safe for further investigation.

### rhACE2 patch protects against hypoxia-induced CM apoptosis

**Fig. 4:**
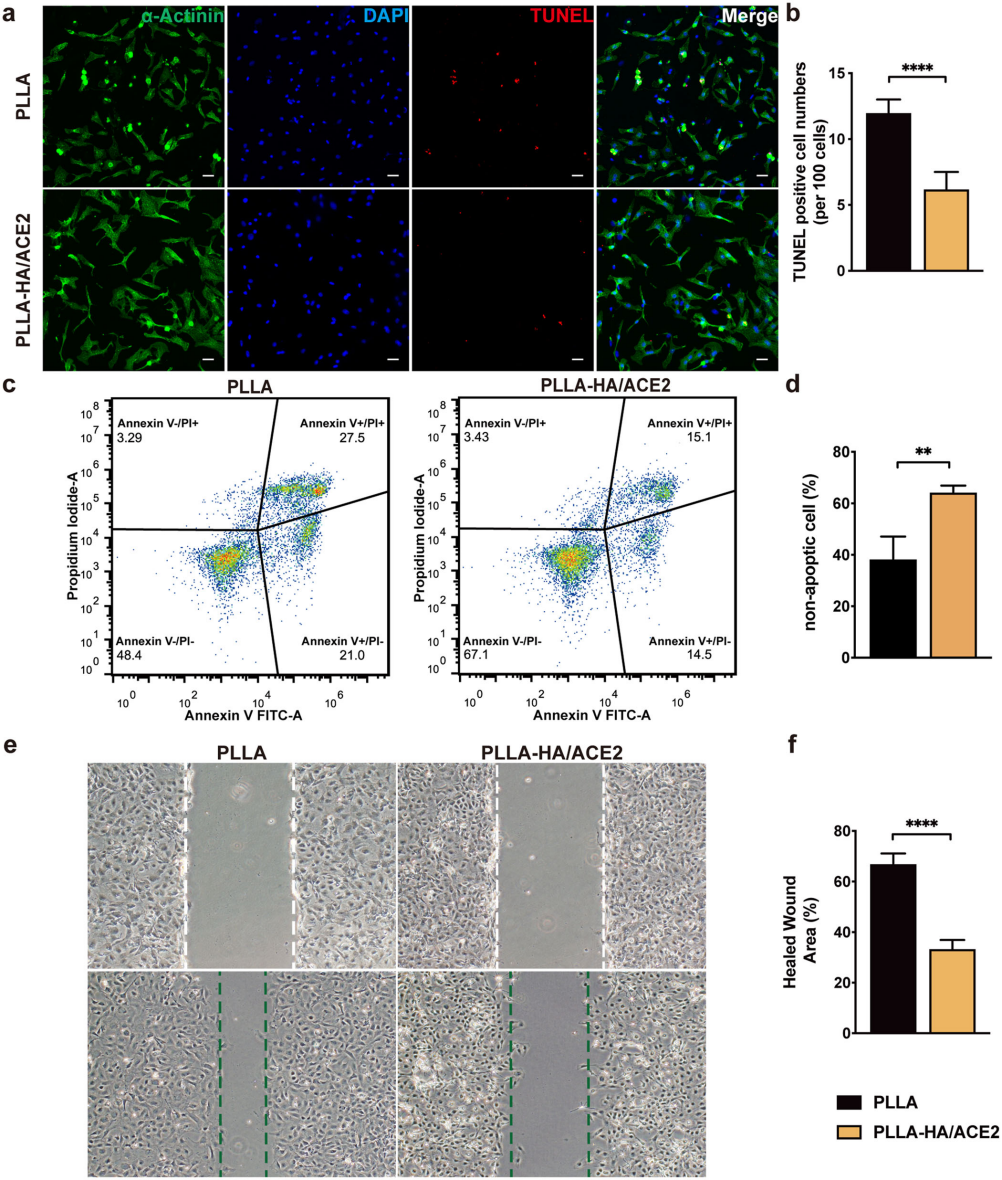
Effects of rhACE2 patch on neonatal rat cardiomyocytes (NRCMs) and cardiac fibroblasts in vitro. **a** Representative immunofluorescence staining of NRCMs exposed to 6 h hypoxia and cocultured with different patches was observed with α-actinin antibody (green), DAPI (blue), and TUNEL (red). Scale bars represented 50 μm. **b** Statistical analysis of TUNEL-positive cell counts per 100 cells. *n* = 5/group. **c** Representative flow cytometric Annexin V FITC-A vs Propidium Iodide-A quad plots of NRCMs after 6 h hypoxia treatment. **d** The percentage of Annexin V−/PI− cell were summarized. *n* = 3/group. **e** Representative wound healing assay image of cardiac fibroblasts cocultured with different patches after 24 h. **f** Statistical analysis of healed wound area difference between two groups. *n* = 5/group. Data were represented as the mean ± SEM. Two group comparisons were analyzed by the unpaired Student’s *t*-test; **P < 0.01, ****P < 0.0001.

An in vitro efficacy assay was performed, in order to confirm whether rhACE2 patch has a protective potency on cardiomyocytes from ischemic damage. NRCMs isolated and cultured, as we stated earlier, were utilized. Hypoxia environment, which lasted 6 h, was introduced to mimic in vivo acute myocardial ischemia. PLLA-HA/ACE2 patch was applied once hypoxia stimuli started and pure PLLA patch was used as a negative control group. α-Actinin was stained as a positive indicator to verify successful isolation of viable NRCMs (Fig. 4a)^25^. Terminal deoxynucleotidyl transferase-mediated deoxyuridine triphosphate nick end labeling (TUNEL) assay was used to detect apoptosis, which illustrated that NRCMs coincubated with PLLA-HA/ACE2 presented less TUNEL-positive cells (12.00 ± 1.00 vs 6.20 ± 1.30 per 100 cell counts, *P* < 0.0001) compared with control group (Fig. 4b). For further revealing how PLLA-HA/ACE2 patch influenced cardiomyocytes survival under hypoxic stress, the Annexin V FITC assay using flow cytometry was also conducted (Supplementary Fig. 1). In PLLA-HA/ACE2 coculture group, the majority of NRCMs (64.4 ± 1.4%) were appeared as viable and non-apoptotic (Annexin V−/PI−) after 6 h hypoxia, compared to only 48.3 ± 5.0% NRCMs in the control group (Fig. 4c, d). A decrease in cardiomyocytes undergoing early apoptosis (Annexin V+/PI−) in treated group was observed. Same trends were also appreciated in the Annexin V+/PI+ gate, which represented a dead or necrotic cell population. Taken all together, these data all supported that rhACE2 patch constructed by PLLA-HA core-sheath structure can prevent cardiomyocyte apoptosis from hypoxic stimuli, of which were critical to cardiac fibrosis reversion after acute MI in vivo.

### rhACE2 patch attenuates cardiac fibroblast migration and proliferation in vitro

To identify in vitro rhACE2 patch effects on cardiac fibroblasts, primary rat neonatal cardiac fibroblast was successfully prepared as previous described for conducting the wound healing study. After wounding, two different patches were seeded with cardiac fibroblasts for 24 h. Compared with the PLLA coculture group, fibroblasts in the PLLA-HA/ACE2 group demonstrated significantly less healing capacity (67.04 ± 4.04% vs 33.41 ± 3.48%, *P* < 0.0001) at 24 h (Fig. 4e, f) after initial wounding. Based on previous observation, it’s safe to conclude that rhACE2 patch can suppress cardiac fibroblast migration and proliferation in vitro.

### Measurement of rhACE2 release and activity in vitro

**Fig. 5:**
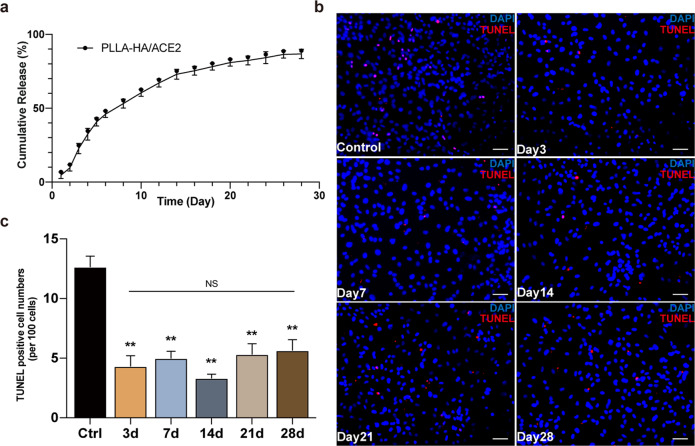
rhACE2 patch sustained-release activity test. **a** In vitro releasing curve of PLLA-HA/ACE2 fibrous membranes. **b** Releasing buffer collected at specified time points during release study was added into culture media of NRCMs undergone 6 h hypoxia. Representative immunofluorescence image of TUNEL (red) and nuclear visualized by DAPI (blue). Scale bars represented 50 μm. **c** Statistical analysis of TUNEL-positive cell counts. *n* = 3/group. Data were represented as the mean ± SEM and analyzed for statistical significance using One-way ANOVA followed by Tukey’s multiple comparison test; NS no significant difference; **P < 0.01 compared to the control group.

The release profile was one of the essential parameters in this study. Only a sustained release of active rhACE2 in situ guaranteed successful establishment of a AngII regulation electrospun fibrous patch niche^26^. The rhACE2 concentrations of releasing buffer from different time points were detected by ELISA. Monitoring of the rhACE2 concentration results demonstrated an early burst release peak of 22.6 ± 3.49% within 3 days, continued with a gradual and continuous release pace for 28 days (Fig. 5a). The overall amount of released rhACE2 after 4 weeks was about 86.8 ± 3.21% of initial loaded amount. This result from the in vitro release assay exhibited the sustained-release capability of our micro-sol electrospun fibrous patch, but the bioactivity of rhACE2 in these releasing buffers cannot be verified by ELISA. In order to determine whether rhACE2 patch preserve its enzymatic activity, these rhACE2 releasing buffers collected from different previous specified timepoints were added into culture media of NRCMs before they underwent hypoxia for 6 h. And the apoptosis status of CMs after hypoxia treatment were identified by TUNEL assays (Fig. 5b). As the quantification analysis showed all releasing buffers from five chronological timepoints demonstrated significantly less TUNEL-positive cell percentage when compared with control group, and no significant difference was observed among releasing buffer groups (Fig. 5c). These results indicated a protection effect for hypoxia-induced cardiomyocytes apoptosis from the rhACE2 patch up to 28 days, proving the electrospun fibrous patch can theoretically create a counter AngII niche in vivo.

### rhACE2 patch improve post myocardial infarction left ventricular dysfunction in vivo

**Fig. 6:**
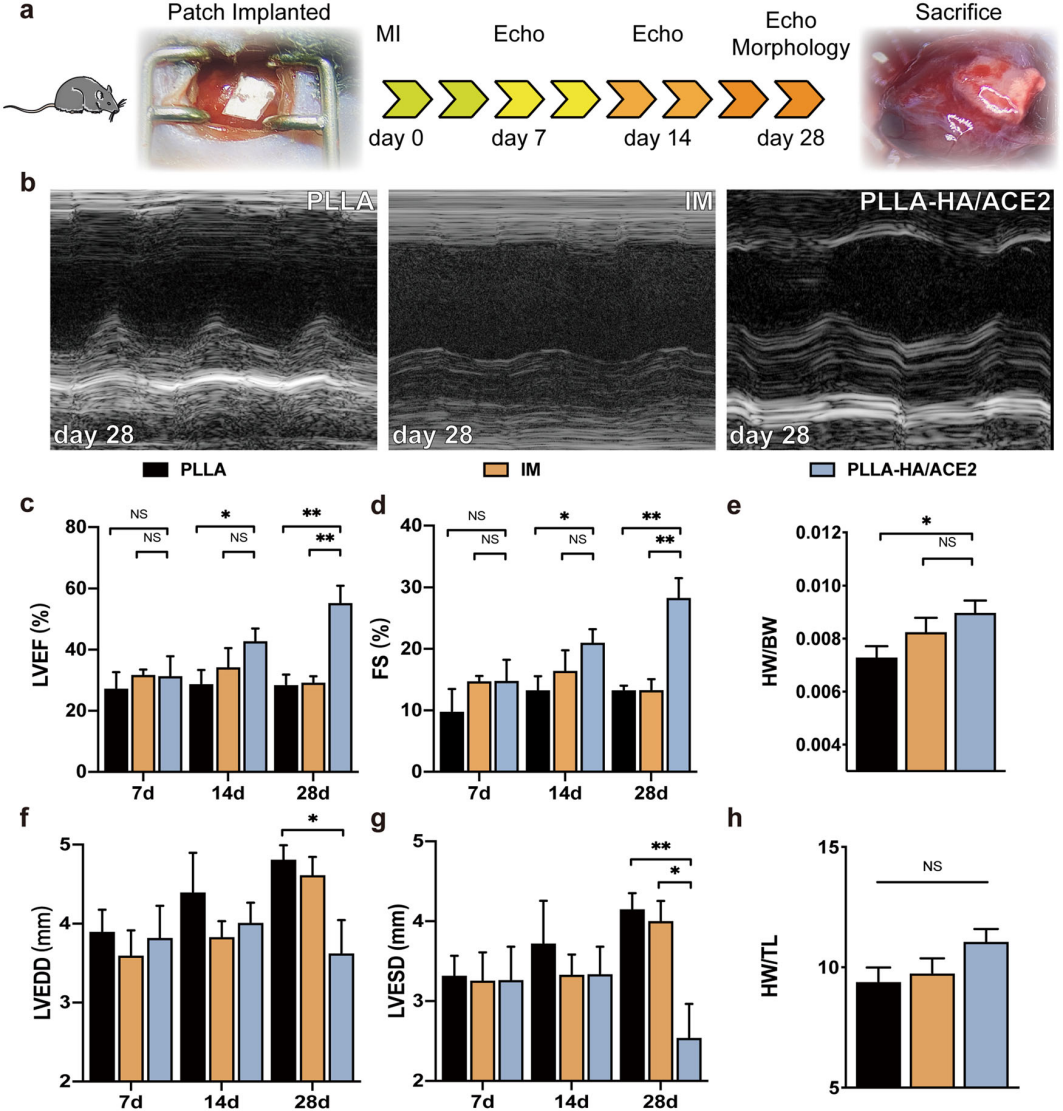
rhACE2 patch preserve left ventricular function after acute myocardial infarction in vivo. **a** The animal research timeline design with images represented the acute MI model and successful implantation of rhACE2 patch. Echo, echocardiography. **b** Representative M-mode parasternal long axis view of left ventricle echocardiographic images of different groups at day 28 after LAD coronary artery ligation. Statistical analysis of left ventricular ejection fraction (**c**), shortening fraction (**d**), heart weight/body weight ratio (**e**), LV end-diastolic diameter (**f**), LV end-systolic diameter (**g**) and heart weight/tibial length (**h**) determined by echocardiography obtained from PLLA, intramyocardial injection (IM) and PLLA-HA/ACE2 treatment group at day 7, day 14 and day 28 after operation. *n* = 5/group. Data were represented as the mean ± SEM and analyzed for statistical significance using two-way ANOVA followed by Tukey’s multiple comparison test; NS no significant difference, *P < 0.05, **P < 0.01.

To confirm our theoretically cardiac fibrosis reversion and post-MI remodeling restriction function of the patch, mice MI model was established by surgical exposed and permanent LAD coronary artery ligation (Fig. 6a). A previously sterilized rhACE2 patch or PLLA patch tailored to 3 × 3 mm was applied onto the heart immediately after ligation. The patch was securely attached to the heart without any additional adhesive or stitch and remained in place after 4 weeks, attributed to an electrostatic effect. And for the intramyocardial injection (IM) group, 0.2 μg of rhACE2 was injected in three different sites around the infarction area using a micro syringe shortly after LAD ligation.

Serial 2-dimensional motion-mode echocardiography was performed at several time points, including day 7, day 14, and day 28 post-MI to determine cardiac function among groups (Fig. 6b). At 1 week after surgery, despite all groups resulted in a markedly depressed left ventricular function. Both IM and rhACE2 patch group showed a trend of better systolic function preservation compared to PLLA group, reflected by left ventricular ejection fraction (PLLA: 27 ± 11%, IM: 31 ± 3%, PLLA-HA/ACE2: 31 ± 14%) and fractional shortening (PLLA: 10 ± 8%, IM: 15 ± 2%, PLLA-HA/ACE2: 15 ± 7%) (Fig. 6c, d). At the same time, no obvious difference was observed in systolic (PLLA: 3.33 ± 0.48 mm, IM: 3.27 ± 0.77 mm, PLLA-HA/ACE2: 3.27 ± 0.91 mm) or diastolic (PLLA: 3.90 ± 0.54 mm, IM: 3.60 ± 0.70 mm, PLLA-HA/ACE2: 3.83 ± 0.89 mm) ventricular chamber diameters (Fig. 6f, g). Same tendency was noticed at 2 weeks post-MI, except rhACE2 patch group demonstrated better systolic function recovery compared with IM group. This phenomenon became more evident overtime. At 4 weeks after initial intervention, the rhACE2 patch group showed significantly better recovery both in systolic function represented by left ventricular ejection fraction (PLLA: 28 ± 7%, IM: 29 ± 4%, PLLA-HA/ACE2: 55 ± 12%) and cardiac structure, either reflected by end-systolic diameter (PLLA: 4.16 ± 0.43 mm, IM: 4.01 ± 0.55 mm, PLLA-HA/ACE2: 2.55 ± 0.94 mm) or end-diastolic diameter (PLLA: 4.82 ± 0.39 mm, IM: 4.62 ± 0.50 mm, PLLA-HA/ACE2: 3.63 ± 0.93 mm), compared to other groups. Similar outcome was observed in the heart weight, hearts from rhACE2 group were heavier than others even after adjusted for body weights (Fig. 6e) or tibial lengths (Fig. 6h). It was worth notice that only the rhACE2 patch group provided sustained anti-remodeling effects during whole timespan and prevented development of heart failure at the end. While IM rhACE2 initially presented some therapeutic effects, it can’t maintain long enough to achieve a clinical meaningful endpoint, because of the short rhACE2 half-life. Such phenomenon provided support to our anticipation to develop a cardiac patch, which can prevent remodeling and chronic heart failure after MI by releasing rhACE2 in-situ continuously and providing a counter AngII niche.

### rhACE2 patch attenuates MI-induced cardiac remodeling

**Fig. 7:**
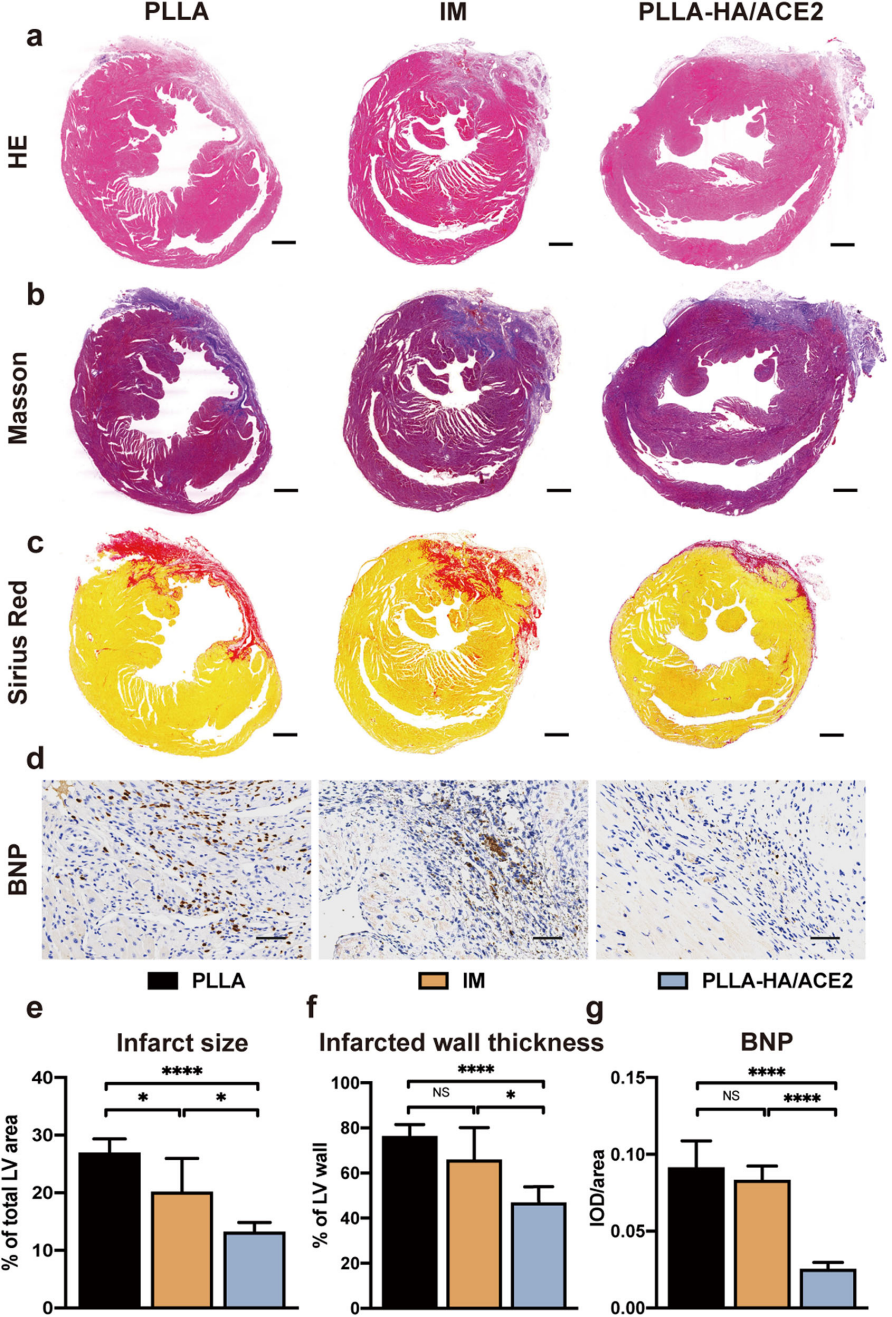
Histological analysis of rhACE2 patch effect on MI-induced cardiac remodeling. H&E stained section (**a**), Masson’s trichrome staining (**b**) and Sirius Red staining (**c**) of mice hearts at low magnification after heart was obtained 28 days after MI. Scale bars represented 500 μm. **d** Brain natriuretic peptide (BNP) immunohistochemical staining of hearts from PLLA, intramyocardial injection (IM) and PLLA-HA/ACE2 treatment group 28 days after MI. Scale bars represented 40 μm. Morphometric parameters including the percentage of infarct size percentage of total LV area (**e**) and the percentage of infarcted thickness of LV anterior wall (**f**) were measured from the Sirius Red stained slides via ImageJ software. *n* = 5/group. **g** The integrated optical density (IOD)/area ratios of BNP were quantified from the immunohistochemical stain by Image-Pro Plus software. *n* = 5/group. Data were represented as the mean ± SEM and was analyzed for statistical significance using one-way ANOVA followed by Tukey’s multiple comparison test; NS no significant difference, *P < 0.05, ****P < 0.0001.

Histological evaluations were performed to further assess the extent of fibrosis and remodeling after mice sacrificed and hearts obtained. In the low magnification field, a striking difference in infarction scar expansion between rhACE2 patch group and others was observed (Fig. 7a). As Masson trichrome staining images demonstrated, apparent fibrosis of infarcted area accompanied with collagen deposition and obvious ventricular thinning were found in the pure PLLA group 28 days after MI (Fig. 7b). By contrast, both IM and rhACE2 patch groups exhibited better preservation of left ventricle structures with only traces of fibrosis and no sign of eccentric ventricle hypertrophy. These Sirius Red staining sections further verified that rhACE2 patch can effectively attenuate myocardial fibrosis by restricting fibrosis within epicardial region and prevent adverse ventricular remodeling (Fig. 7c). Morphometric analysis of all sections also revealed that rhACE2 patch showed a significantly better effect in preventing infarction expansion in a long term compared to other groups (PLLA: 27.0 ± 2.32%, IM: 20.2 ± 5.73%, PLLA-HA/ACE2: 13.3 ± 1.58%), which was probably due to its sustained-release niche effects (Fig. 7e). In terms of infarction thickness, a statistically significant protective effect in reversing transmural cardiac fibrosis after ischemia (PLLA: 76.5 ± 5.01%, IM: 66.1 ± 13.99%, PLLA-HA/ACE2: 47.1±6.64%) was observed, only when rhACE2 was administered by the patch we tested here (Fig. 7f).

Brain natriuretic peptide is a hormone secreted by cardiomyocytes when ventricle is stretched during a volume overload status, which thus is used as a well acknowledged biomarker of heart failure^27^. BNP was tested in myocardial tissue by immunohistochemical staining to reveal whether heart failure was doomed due to post-MI remodeling (Fig. 7d). All cardiomyocytes secreting BNP were stained as brown. As we have expected, the rhACE2 patch was the only intervention group in which BNP wasn’t pathologically upregulated in myocardium. Quantitative analysis calculating integrated optical density (IOD)/area ratio by Image Pro Plus confirmed the results, showing either PLLA or IM group expressed significantly more BNP in tissues (PLLA: 0.09 ± 0.017, IM: 0.08 ± 0.008, PLLA-HA/ACE2: 0.02 ± 0.004) (Fig. 7g), which represented these groups enduring worse volume stress and developing heart failure after large scale cardiac fibrosis. In the meantime, application of rhACE2 patch successfully reverted massive fibrosis and halted adverse remodeling. Taken altogether, all the animal histological experiment data indicated the prevention of ischemic heart failure from developing by our rhACE2 patch. And all histology data were consistent with previous in vitro NRCMs and cardiac fibroblasts experiment results and cardiac function results detected by echocardiography.

## Discussion

After MI, heart suffers from fibrosis, adverse remodeling, ventricle dysfunction and finally resulted in heart failure^28^. Compared with regular remodeling prevention strategy like oral neurohormonal blockade agents, reversing myocardial fibrosis by an AngII induction transformation niche shaping patch is a comparatively convenient, natural and effective approach^29,30^. In this study, the development and efficacy of the rhACE2-loaded fibrous patch were explicated. Enlightened by the localized paracrine expression modus of ACE2^31,32^, we brought forward a in situ renin–angiotensin–aldosterone axis regulation patch. As a relatively accessible and cost-efficient synthetic fibrous membrane, the rhACE2 patch was designed to release bioactive rhACE2 continuously, for further inducing local AngII to transform into Ang1–7, reversing post-MI fibrosis and preventing ventricle remodeling.

First step of our experiments was to design and integrate biomaterial structures. ACE2 is a vigorous anti-fibrotic protein, which plays a key negative regulation role in neurohormonal overactivation. However, subjected to the short terminal half-life of rhACE2 and the dependence of sufficient local retention, effective dosage of rhACE2 requires repetitive administration, which can be potentially harmful^33^. Therefore, a local drug carriage system for durable release of rhACE2 is warranted. As previous study reported^34^, HA hydrosol particles served as a storage vehicle to protect rhACE2 from degradation in organic solvent by controlling hydrophilic drug release from hydrophobic polyester fibers. Additionally, HA inside the core is a trustful biomaterial which can be utilized in extracellular matrix with some anti-inflammatory and antibiosis effect^35^. Thus, HA-sol was selected as the rhACE2 delivery conveyor. The viscosity of the PLLA solution increase faster than the inner HA-sol core, due to water volatilized slower than the organic solvent^36^. So, through the spinning process during synthesis, the HA-sol shift and stretch inside the PLLA sheath forming a core-sheath structure. Finally, the core-sheath structure with the sol-coated rhACE2 particles inside the core and the PLLA wrapping around as the outer sheath was successfully constructed using the micro-sol electrospinning technology.

To prove the fibrous patch can theoretically shape an anti-fibrotic niche^37,38^, we verified its biocompatibility, protein releasing trait and bioactivity to primary cardiac cells. Safety of the rhACE2 patch was reassured by our NRCMs coculture and CCK-8 study. The stability and therapeutic potency of rhACE2 patch within a 28-day timespan were confirmed through reducing NRCMs hypoxia-induced apoptosis by its releasing buffer collected from different timepoints. Using various methods including immunofluorescence and flowcytometry, rhACE2 patch demonstrated potential to improve NRCMs survival and inhibit cardiac fibroblasts proliferation, which both lead to the remodeling reversion trait. Unlike former studies using ACE2 gene overexpression^39^ or a daily intraperitoneal injection^7^ to exert its benefits, this study integrated therapeutic but volatile rhACE2 into a synthetic micro-sol electrospun fiber to create an easy-to-use, effective and stable rhACE2 cardiac patch.

As the motif of this study, reversion of fibrosis and prevention of cardiac remodeling by the rhACE2 patch were eventually observed in animal MI models. Ejection fraction tumbled and remained persistent low in the PLLA control group, followed with enlargement in left ventricle diameter. These results were also in accordance with morphology, as histological analysis depicted a dilated heart with severe transmural fibrosis and no sign of viable myocytes at infarcted area, showing an evident remodeling pathology. For rhACE2 intramyocardial injection group, some protection effect can be noticed in early days after MI, reflecting by better ejection fraction and less dilated ventricle similar as the rhACE2 patch group. However, this favorable trend can no longer be observed 4 weeks after MI, suggesting single IM administration can’t provide adequate protection against ischemia induced remodeling. This phenomenon probably is due to degradation and extrusion of injected agents during continuous dynamic cardiac muscle contraction, impeding formation of a counter remodeling niche. It is conceivable that both these groups end up in unfavored prognosis^40^. Only in the rhACE2 patch group, a definite recovery in ventricle function and morphology was observed. Meanwhile, less BNP immunohistochemical staining further morphologically indicated less volume overload and ventricle wall tension^41^.

Taken together all cellular and animal experiment results, the rhACE2 patch shaped an AngII induce transformation niche, which reduced post-MI cardiomyocytes apoptosis, inhibited fibroblasts activation and prevented adverse remodeling from progression. AngII expression increased excessively within myocardium after MI, which promoted cardiac fibrosis and initiated ventricular remodeling^42^. The rhACE2-electrospun patch provided a durable release of rhACE2 from the fibers core, which cleaved exceeding AngII to Ang1–7. In this way, the rhACE2 patch utilized local AngII, as natural substrates, to form a post-MI counter-fibrosis niche, turning enemies into friends through the induction transformation process. Additionally, micro-sol electrospun membrane, which possess a porous structure, could permit endogenous cytokines and inflammatory cells recruited to pass gradually. Also, the electrospun fibrous patch with proper stress-strain property and electrostatic energy could spontaneously attached onto the epicardium, providing a physical support to the damaged wall in prevention of a protruding ventricle aneurysm.

A classic cardiac patch focus on matrix of biological origin and cell therapies cannot satisfy the economic, storage and consistency requirement in the real world^15–17^. Therefore, we proposed a synthetic micro-sol electrospun rhACE2 patch focusing on neurohormonal blockade. With affordable costs and storage conditions, the synthetic patch resolved two major obstacles to rhACE2 clinical application, which include low cardiac retention and short half-life. It can be potentially implanted during thoracic surgery like coronary artery bypass grafting to prevent ischemic cardiomyopathy in future.

But as a biomaterial aiming for clinical improvements, more studies will be required. First, the proportion of rhACE2, the microstructure such as the diameter and tension of fibers and the thickness and area of the patch may decide different therapeutic outcomes, which demand optimization by more carefully designed experiments. Second, further validation of rhACE2 patch efficacy on cardiomyocytes derived from induced pluripotent stem cells of patients with ischemic heart diseases is scheduled to conduct next. In addition, in vivo studies with porcine MI models in a longer timespan than 4 weeks should be investigated to broaden the clinical application.

In summary, focusing on a cost-effective synthetic cardiac patch and resolving limitations in rhACE2 administration, a rhACE2-electrospun fibrous patch niche was established in our study, which could form an Ang II induction transformation niche for fibrosis reversion and remodeling prevention by micro-sol electrospinning technologies. The protective effects were characterized by reducing cardiomyocytes apoptosis under hypoxic stress and inhibiting fibroblasts proliferation, which represented a cellular level mechanism. Furthermore, ventricular function and structure recovery were confirmed in MI mice models, reflecting by improved left ventricular ejection fraction, normal ventricle structure and less fibrosis. Accordingly, the rhACE2 patch niche provided a feasible method for clinical treatment of ischemic heart failure.

## Methods

### Materials

PLLA (Mw = 100 kDa, Mw/Mn = 2.16) was obtained from Jinan Daigang Co. (China). Fermentation-derived HA (sodium salt, Mw = 0.5 Mda) was acquired from Yuancheng Technology Co. (China) and applied without further purification. Human ACE2 (18-652) recombinant protein (rhACE2, #85054) was purchased from Cell Signaling Technology (USA). rhACE2 contains necessary enzymatic activity associated protein structure and its effectiveness in mice and rats had been previously tested^7^, so it was selected for both our cell and animal studies. Dulbecco’s modified Eagle’s medium (DMEM), fetal bovine serum, trypsin and penicillin/streptomycin were purchased from Gibco (USA). All other reagents, if not otherwise specifically mentioned, were obtained from GuoYao Regents Co. (China).

### Preparation of electrospun patches

The electrospinning technique performed here was described previously^43^. Briefly, the electrospinning solution was contained by a 10 mL syringe equipped with a steel blunt needle with inner diameter of 0.9 mm. A metal clip which was connected to the DC high-voltage power supply obtained from Tianjin Dongwen High-voltage Power Supply Co. (China) was attached at the end of needle. From the tip of the needle, an electrically grounded aluminum foil was placed at a specific distance to collect electrospun fibers. For injection rate control, precision pumps (LSP02-1B) acquired from Baoding Longer Precision Pump Co. (China) were used.

For the electrospun micro-sol membranes, 12 mg HA was dissolved in 988 μL distilled water to make 1.2 wt% HA hydrosol. Then, 10 μL rhACE2 (100 μg/mL) was mixed into 50 μL HA hydrosol to eventually form uniform 1% HA-rhACE2 hydrosol. Next, the rhACE2-loaded HA hydrosol was mixed with a solvent mixture containing 3.03 mL dichloromethane (DCM) and 0.01 g Span-80. In order to form a water-in-oil emulsions containing uniformly micro-sol particles, the mixture was stirred at a high speed. Lastly, 0.5 g PLLA and 2.11 mL N, N-dimethylformamide (DMF) were dissolved in the emulsion to obtain micro-sol electrospun solution. And the pure PLLA electrospun solution used as control was formed by mixing and stirring 0.5 g PLLA, 3.03 mL DCM and 2.11 mL DMF. The electrospun parameters were 60 μL/min for flow rate, 15–20 kV for applied voltage, 15 cm for collecting distance.

All electrospun membranes were vacuum-dried overnight to completely remove any residual solvent before application.

### Characteristics of patches

First, the size distribution of HA/rhACE2 micro-sol particles dispersed in DCM was analyzed by dynamic light scattering (DLS, Malvern, Nano-ZS90, UK). For the purpose of observation of the microstructural morphology, patch samples of appropriate size were mounted on the SEM sample stub via conductive tapes, followed by gold sputter coating procedure for 45 s (Quorum Technologies, SC7620, UK). After preparation procedures, a scanning electron microscope (SEM, Hitachi, S-4800, Japan) was used to observe the surface morphology at an accelerating voltage of 10 kV. Image J was used to analyze the average diameter of inner and outer structure by measuring a total of 200 random fibers in five separate SEM images. The transmission electron microscope (TEM, Hitachi, HT7700, Japan) was used to verify the inner structure of individual fibers at a voltage of 120 kV. Water contact angle measurement was performed after 10 s of sessile drop on a contact angle meter VCA optima Surface Analysis System (WCA, Data Physics Corporation, DSA25S, Germany) to evaluate the wettability. For mechanical parameters, strips (15.0 × 3.0 × 0.1 mm) were prepared by a mold before tensile test. Load-deformation data were measured at a speed of 2.5 mm/min using mechanical testing machine (Shanghai Hengyi Precision Instruments Co., China).

### Cell viability assay and apoptosis detection

For evaluation of cell viability, 1 mL cell suspension (1 × 10^5^ cells/mL) was seeded into each well. Live cells were stained by Calcein AM (Beyotime, China) and dead cells were marked using Propidium Iodide (Beyotime, China). After 3 days of coculture, cells were stained with Calcein AM (2 μg/mL) + Propidium Iodide (1 μg/mL) working solution and incubated for 30 min at 37 °C. Cell counting kit-8 (CCK-8, Dojindo, Japan) was mixed with culture medium in a volume ratio of 10% at desired time timepoints (1, 2, and 3 days) to quantify cell viability. After 4 h incubation, 100 μL of the mixed solution was transferred into a 96-well plate. Absorbance at 450 nm was measured using a Microplate reader (model 680, Bio-Rad, USA).

In vitro cell apoptosis was detected by terminal deoxynucleotidyl transferase-dUTP nick end labeling (TUNEL) assay kit provided by Invitrogen (USA) and measured by Flow Cytometry using Annexin V FITC apoptosis detection kit purchased from BD Biosciences (USA). Both tests were conducted following the manufacturer’s protocol. Stained cells were photographed by automated fluorescence microscope (Carl Zeiss Inc., Zeiss Axiovert 200, USA). The number of apoptotic cells was counted using ZEN 2.3 software (Zeiss). The apoptosis ratio was calculated by the number of TUNEL-positive cells per 100 cells in 5 different representative microscopic fields. For flow cytometry, the treated cell samples were analyzed using BD FACSCalibur flowcytometry (USA) after stained as protocol required. The amounts of early and late apoptosis were determined as the percentage of Annexin V FITC positive/Propidium Iodide negative or Annexin V FITC positive/Propidium Iodide positive cells, respectively.

### Neonatal rat cardiomyocytes culture and hypoxia model

Primary NRCMs were isolated as previously reported^44^. Briefly, 1-day-old Sprague-Dawley rats were euthanized, and the hearts were collected. The ventricles were thoroughly minced and digested with a digestion solution containing 0.05% collagenase type 2. Cell suspension were collected, centrifuged, resuspended in the culture medium and plated for 1 h under standard culture conditions, to allow fibroblasts separated from cardiomyocytes. Cell suspension which mainly containing NRCMs was collected and plated onto culture dishes with complete medium. Successful isolation was confirmed by beating morphology and immunostaining after 24 h. To establish the hypoxia model, we moved the cells into an anaerobic incubator containing 94% N_2_, 5% CO_2_ and 1% O_2_, after replacing the culture medium with serum-free, glucose-free DMEM. Unless otherwise indicated, NRCMs were subjected to hypoxia for 6 h.

### Protein release and activity study

For release assay, rhACE2 fibrous patch (10 mg which contained 20 ng rhACE2 in theory) were soaked in10 mL PBS containing 1% bovine serum albumin (BSA) in 50 mL centrifuge tubes. The tubes were then placed in a thermostatic shaker at 37 °C set to 100 cycles/min. At designated time points (day 1, 2, 3, 4, 5, 6, 8, 10, 12, 14, 16, 18, 20, 22, 24, 26, 28), releasing buffer was collected from the centrifuge tube, stored at −20 °C before further test and replaced with 10 mL fresh 1% BSA. The amount of released rhACE2 from the fibrous patch was quantified by human ACE2 ELISA kit (Cusabio, USA). According to the initial amount of rhACE2 encapsulated into the patch, the release curve was depicted. To verify rhACE2 bioactivity in the releasing buffers, 4 × 10^4^ cells/mL were plated in 24-well plates. And 1 mL of releasing buffer collected previously was added to each well with the hypoxia medium before moving the plate into the anaerobic incubator. After the 6 h hypoxia stress, a TUNEL assay was conducted as previously described to assess NRCMs apoptosis status.

### Animal studies and myocardial infarction model

All animal experiment protocols were approved by the Laboratory Animal Ethics Committee of Ruijin Hospital. Mice were obtained from Model Animal Research Center of Nanjing University. All mice were housed under the Experimental Medical Research Center of Ruijin Hospital with a standard 12-h light-dark cycle and free access to food and water.

Permanent ligation of the LAD coronary artery was performed on 8-week-old C57BL/6 male mice, as we reported before^44^. Briefly, mice were anesthetized with isoflurane and ventilated mechanically. A left thoracotomy was conducted at the fourth intercostal rib space. The heart was exposed to ligate the LAD permanently using a 7-0 silk suture. Fibrous patches were implanted directly onto the infarction zone after the ligation. As the intramyocardial injection group, 0.2 μg of rhACE2 (0.1 μg/μL) was injected at five different points around the infarction zone by a 30G micro syringe (Hamilton, USA).

### Echocardiography

Echocardiography was performed to assess ventricle function, including LV ejection fraction, LV fractional shortening, cardiac output, LV end-diastolic diameter, LV end-systolic diameter and LV mass at day 7, day 14 and day 28 after MI using a Vevo 2100 instrument (FUJIFILM Visual Sonics, Canada) equipped with a MS-400 transducer. M-mode images were captured from the parasternal long axis of the heart at the papillary muscle level.

### Histological analysis

Hearts were fixed in 4% paraformaldehyde, embedded in paraffin, and dissected into 5-μm-thick serial transverse sections at the papillary muscle levels. Hematoxylin and eosin staining, and Masson’s trichrome staining were used to evaluate heart tissue morphology. Sirius red staining was performed to visually assess cardiac fibrosis extents. Immunohistochemical staining was conducted using anti-BNP antibody (1:500) (Abcam, ab239510) for 24 h at 4 °C and horseradish peroxidase-linked secondary antibody (1:500) (Abcam, ab97023) for 60 min at room temperature. And further immunohistochemistry steps were conducted as previously reported methods. Images were captured by a microscope (BX61TRF, Olympus, Japan) and quantified using Image J software (Ver 1.51, National Institutes of Health) and Image-Pro Plus 6.0 software (Media Cybernetics Inc.).

### Statistical analysis

Normally distributed data were expressed in the form of means ± standard error of the mean. Statistical analysis was evaluated by One-way ANOVA followed by Tukey’s multiple comparison test or Student’s *t*-test as appropriate using GraphPad Prism 8 (USA) for between groups differences, unless otherwise stated. The value of significance level α was defined as 0.05.

### Reporting summary

Further information on research design is available in the Nature Research Reporting Summary linked to this article.

## Supplementary information

Supplementary Information

Reporting Summary

## Data Availability

The data that support the findings of this study are available from the corresponding author upon reasonable request.
