# Supplementary material for: Myocardial fibrosis reversion via rhACE2-electrospun fibrous patch for ventricular remodeling prevention
Source: NPJ Regen Med. 2021 Aug 10;6:44. doi: 10.1038/s41536-021-00154-y (PMC8355140; doi:10.1038/s41536-021-00154-y)
Supplement: Supplementary file 1 — Supplementary Information [file 41536_2021_154_MOESM1_ESM.pdf]

Supplementary Information

Myocardial fibrosis reversion via rhACE2-electrospun fibrous patch for ventricular remodeling prevention

Zeping Qiu, Jingwen Zhao, Fanyi Huang, Luhan Bao, Yanjia Chen, Ke Yang, Wenguo Cui, Wei Jin

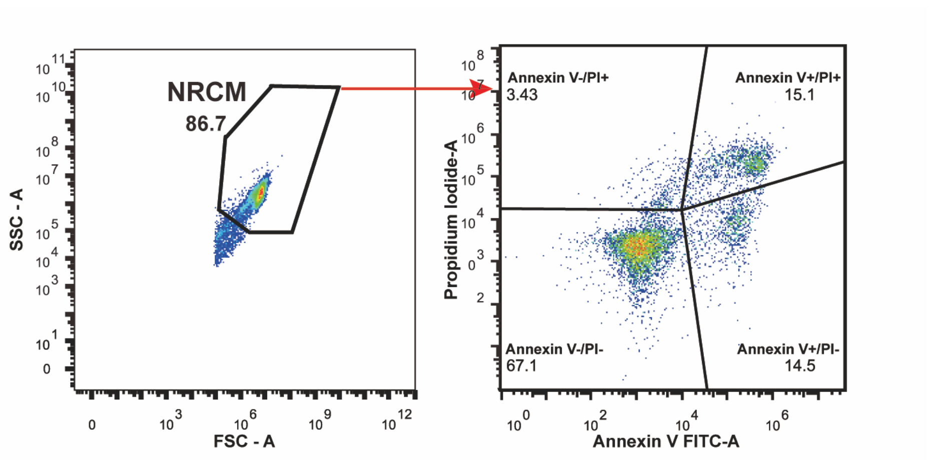

Supplementary Figure1. Sorting Strategy for the Annexin V FITC assay relate to Figure 4c. NRCM, neonatal rat cardiomyocytes; FSC-A, forward scatter-area and SSC-A, side scatter-area.
