# Supplementary material for: Myocardial fibrosis reversion via rhACE2-electrospun fibrous patch for ventricular remodeling prevention
Source: NPJ Regen Med. 2021 Aug 10;6:44. doi: 10.1038/s41536-021-00154-y (PMC8355140; doi:10.1038/s41536-021-00154-y)
Supplement: Supplementary file 2 — Reporting Summary [file 41536_2021_154_MOESM2_ESM.pdf]

nature research

Corresponding author(s): Wei Jin

Last updated by author(s): May 13, 2021

Reporting Summary

Nature Research wishes to improve the reproducibility of the work that we publish. This form provides structure for consistency and transparency in reporting. For further information on Nature Research policies, see our Editorial Policies and the Editorial Policy Checklist.

Statistics

For all statistical analyses, confirm that the following items are present in the figure legend, table legend, main text, or Methods section.

n/a	Confirmed	
☐	☒	The exact sample size (*n*) for each experimental group/condition, given as a discrete number and unit of measurement
☐	☒	A statement on whether measurements were taken from distinct samples or whether the same sample was measured repeatedly
☐	☒	The statistical test(s) used AND whether they are one- or two-sided<br>*Only common tests should be described solely by name; describe more complex techniques in the Methods section.*
☐	☒	A description of all covariates tested
☐	☒	A description of any assumptions or corrections, such as tests of normality and adjustment for multiple comparisons
☐	☒	A full description of the statistical parameters including central tendency (e.g. means) or other basic estimates (e.g. regression coefficient) AND variation (e.g. standard deviation) or associated estimates of uncertainty (e.g. confidence intervals)
☐	☒	For null hypothesis testing, the test statistic (e.g. *F*, *t*, *r*) with confidence intervals, effect sizes, degrees of freedom and *P* value noted<br>*Give P values as exact values whenever suitable.*
☐	☒	For Bayesian analysis, information on the choice of priors and Markov chain Monte Carlo settings
☐	☒	For hierarchical and complex designs, identification of the appropriate level for tests and full reporting of outcomes
☐	☒	Estimates of effect sizes (e.g. Cohen's *d*, Pearson's *r*), indicating how they were calculated

Our web collection on statistics for biologists contains articles on many of the points above.

Software and code

Policy information about availability of computer code

Data collection	Echocardiography was conducted using Vevo 2100 instrument (FUJIFILM Visual Sonics, Canada).
Data analysis	Zen 2.3 software (Zeiss) was used to count apoptotic cells.<br>Image J software (Ver 1.51, National Institutes of Health) and Image-Pro Plus 6.0 software (Media Cybernetics Inc.) were used to analyze histology.<br>Statistic analysis were performed using GraphPad Prism 8 (USA).

For manuscripts utilizing custom algorithms or software that are central to the research but not yet described in published literature, software must be made available to editors and reviewers. We strongly encourage code deposition in a community repository (e.g. GitHub). See the Nature Research guidelines for submitting code & software for further information.

Data

Policy information about availability of data

All manuscripts must include a data availability statement. This statement should provide the following information, where applicable:

- Accession codes, unique identifiers, or web links for publicly available datasets
- A list of figures that have associated raw data
- A description of any restrictions on data availability

The data that support the findings of this study are available from the corresponding author upon reasonable request.

Field-specific reporting

Please select the one below that is the best fit for your research. If you are not sure, read the appropriate sections before making your selection.

☒ Life sciences ☐ Behavioural & social sciences ☐ Ecological, evolutionary & environmental sciences

For a reference copy of the document with all sections, see nature.com/documents/nr-reporting-summary-flat.pdf

Life sciences study design

All studies must disclose on these points even when the disclosure is negative.

Sample size	All animal experiment protocols were approved by the Laboratory Animal Ethics Committee of Ruijin Hospital. Sample size was chosen to provide sufficient replications, while harm less animals.
Data exclusions	No data were exculded.
Replication	All findings were successfully replicated in repeat experiments.
Randomization	8-week-old C57BL/6 male mice were used in the study, no randomization procedure required.
Blinding	The operator who performed LAD coronary artery ligation or echocardiography was blinded to group allocation.

Reporting for specific materials, systems and methods

We require information from authors about some types of materials, experimental systems and methods used in many studies. Here, indicate whether each material, system or method listed is relevant to your study. If you are not sure if a list item applies to your research, read the appropriate section before selecting a response.

Materials & experimental systems

n/a	Involved in the study	
☐	☒	Antibodies
☒	☐	Eukaryotic cell lines
☒	☐	Palaeontology and archaeology
☐	☒	Animals and other organisms
☒	☐	Human research participants
☒	☐	Clinical data
☒	☐	Dual use research of concern

Methods

n/a	Involved in the study	
☒	☐	ChIP-seq
☐	☒	Flow cytometry
☒	☐	MRI-based neuroimaging

Antibodies

Antibodies used	The anti-BNP antibody was purchased from Abcam (ab239510). The horseradish peroxidase-linked secondary antibody was purchased from Abcam (ab97023).
Validation	The anti-BNP antibody was validated by the manufacturer for IHC.<br>The horseradish peroxidase-linked secondary antibody was referenced by multiple publications, including Nat Commun 11:2702 (2020); Cell Rep 27:514-524.e5 (2019).

Animals and other organisms

Policy information about studies involving animals; ARRIVE guidelines recommended for reporting animal research

Laboratory animals	8-week-old C57BL/6 male mice were used.
Wild animals	No wild animals were involved.
Field-collected samples	This study did not involve samples collected from field.
Ethics oversight	All animal experiment protocols were approved by the Laboratory Animal Ethics Committee of Ruijin Hospital.

Note that full information on the approval of the study protocol must also be provided in the manuscript.

Flow Cytometry

Plots

Confirm that:

☒ The axis labels state the marker and fluorochrome used (e.g. CD4-FITC).

☒ The axis scales are clearly visible. Include numbers along axes only for bottom left plot of group (a 'group' is an analysis of identical markers).

☒ All plots are contour plots with outliers or pseudocolor plots.

☒ A numerical value for number of cells or percentage (with statistics) is provided.

Methodology

Sample preparation	Primary neonatal rat cardiomyocytes (NRCMs) were isolated and cultured.
Instrument	BD FACSCalibur flowcytometry (USA) was used to analyze the samples.
Software	Flowjo Ver 10 was used to analyze the flow cytometry data.
Cell population abundance	Cardiomyocytes were gated by Annexin V FITC-A vs Propidium Iodide-A (PI-A) to seperate 4 different populations, including Annexin V – /PI–, Annexin V+ /PI–, Annexin V+ /PI+, Annexin V –/PI+
Gating strategy	Annexin V FITC-A vs Propidium Iodide-A (PI-A)

☒ Tick this box to confirm that a figure exemplifying the gating strategy is provided in the Supplementary Information.
